# Alterations of CCR2 and CX3CR1 on Three Monocyte Subsets During HIV-1/*Treponema pallidum* Coinfection

**DOI:** 10.3389/fmed.2020.00272

**Published:** 2020-06-18

**Authors:** Na Guo, Yongchang Chen, Bin Su, Xiaodong Yang, Qiuyue Zhang, Ting Song, Hao Wu, Cuie Liu, Lifeng Liu, Tong Zhang

**Affiliations:** ^1^Center for Infectious Diseases, Beijing Youan Hospital, Capital Medical University, Beijing, China; ^2^Beijing Key Laboratory for HIV/AIDS Research, Beijing, China; ^3^Department of Dermatology, Beijing Youan Hospital, Capital Medical University, Beijing, China

**Keywords:** HIV-1, syphilis, monocyte subset, CCR2, CX3CR1

## Abstract

HIV-1/*Treponema pallidum* (*T. pallidum*) coinfection has become a global challenge, and three monocyte subsets express varying levels of the chemokine receptors CCR2 and CX3CR1. We recently evaluated the association between monocyte subsets and regulatory T cells in HIV-infected individuals with syphilis. Currently, the dynamic changes of CCR2 and CX3CR1 on monocyte subsets during HIV-1 and syphilis coinfection have not been fully investigated. In this study, cell surface staining was used to explore CCR2 and CX3CR1 expression on three monocyte subsets during HIV-1/*T. pallidum* coinfection. We found that CCR2 densities on the classical monocyte subsets decreased in acute HIV-1 infected (AHI) patients, chronic HIV-1-infected individuals without antiviral therapy (ART) (CHI+ ART–), chronic HIV-1-infected individuals receiving ART (CHI+ART+), rapid plasma reagin-positive (RPR+) individuals, CHI+ ART– plus RPR+ (CHI+RPR+ ART–) individuals, and CHI+ART+ plus RPR^+^ (CHI+RPR+ART+) individuals. CX3CR1 density increased on the three monocyte subsets during HIV-1 and/or *T. pallidum* infection. CX3CR1 density on the intermediate and non-classical monocyte subsets in CHI+ ART– individuals was lower than that in CHI+ART+ individuals, and CX3CR1 density on the three monocyte subsets in CHI+ART+ individuals was higher than that in CHI+RPR+ART+ individuals. Our data provide new insight into the roles of CCR2 and CX3CR1 on three monocyte subsets in HIV-1 and *T. pallidum* pathogenesis.

## Introduction

HIV-1/*Treponema pallidum* (*T. pallidum*) coinfection has become a global challenge among men who have sex with men (MSM) (1). The prevalence of syphilis and HIV-1 coinfection among MSM was 28.7% in Istanbul, Turkey (2). A total of 12.5% of syphilis infected MSM were HIV-positive in 61 cities in China (3).

HIV-1 and *T. pallidum* act synergistically to accelerate transmission and disease progression (4, 5). *T. pallidum* recruits HIV-1 susceptible inflammatory cells, such as activated macrophages, to the infection site (6). Syphilis infection differentially regulates the phenotype and function of gammadelta T cells at different stages of HIV-1 diseases (7). The immunological response to syphilis differs during the course of HIV-1 disease progression (8). *T. pallidum*-specific antibody activity was reduced in HIV-infected patients with syphilis (9). Approximately 12% of serological treatment response failures occur in early and late syphilis infected patients with HIV-1 because of immunosuppression (10), and repeat syphilis infection is likely responsible for asymptomatic infection in HIV-infected patients (11). An increased titer RPR, delay or failure of titer decline, and clinical relapse have been described during the course of syphilis in HIV-1-infected patients (12).

Based on CD14 and CD16 expression, human monocytes have been subdivided into three subsets: classical monocyte subsets (CD14^++^CD16^−^), intermediate monocyte subsets (CD14^++^CD16^+^) and non-classical monocyte subsets (CD14^+^CD16^++^) (13). We recently found that the frequency of the intermediate monocyte subsets in acute HIV-1-infected individuals was significantly higher than that in healthy controls, and the frequency of the intermediate monocyte subsets was inversely correlated with CD4^+^ T cell counts during HIV-1 infection (14). We also found that the frequency of the classical monocyte subsets was higher in syphilis patients than that in HCs and in syphilis/HIV-1 coinfected patients (15).

The intermediate and non-classical monocyte subsets express increased levels of CX3CR1, while classical monocytes express increased levels of CCR2 (16). It was reported that CCR2^+^ monocytes promote colon fibrosis by inhibiting collagen degradation through tissue inhibitor of metalloproteinase (TIMP-1) production in inflammatory bowel disease (IBD) (17). CCR2 on CD14^+^CD16^+^ monocytes can act as a novel biomarker of HIV-1-associated neurocognitive disorders (HANDs) (18). The CCL2/CCR2 axis is linked to viral replication and immune activation during HIV-1 infection, and modulation of this axis may have an impact on HIV disease progression. CX3CR1 is the receptor of fractalkine, which is also known as CX3CL1. The fractalkine/CX3CR1 axis plays an important role in the pathogenesis of many diseases with imbalances of immune response. Interruption of the fractalkine/CX3CR1 axis has been shown to ameliorate murine colitis by regulating monocyte behaviors (19, 20). Until now, the alteration of CCR2 and CX3CR1 expression on three monocyte subsets at different stages of HIV-1 and *T. pallidum* infection has not been fully investigated.

In this study, we investigated the alterations of CCR2 and CX3CR1 on the three monocyte subsets during HIV-1/*T. pallidum* coinfection.

## Materials and Methods

### Study Participants

All subjects in the study provided written consent according to the Declaration of Helsinki. The study was approved by the Beijing Youan Hospital Research Ethics Committee. Seven groups were included in the study: Thirty-four acute HIV-1-infected (AHI, group 1) patients were randomly enrolled from the Beijing PRIMO Clinical Cohort, the cohort was from high-risk HIV-1-negative men who have sex with men (MSM), and the patients in the cohort were tested for HIV-1 antibodies every 3 months. On the basis of laboratory test results, AHI patients were in Fiebig stage III-V (21). Forty-nine male chronic HIV-1-infected individuals were randomly enrolled from the HIV/AIDS clinic of Beijing Youan Hospital, which consisted of 25 chronic HIV-1-infected individuals without ART (CHI+ ART–, group 2) and 24 patients receiving ART (CHI+ART+, group3). Seventeen RPR+ individuals were seropositive in both the TPPA and RPR tests within 1 year (group 4). Forty-six chronic HIV-1-infected individuals were diagnosed with early syphilis: 16 chronic HIV-1-infected individuals without ART patients with syphilis (CHI+RPR+ ART–, group 5) and 30 patients receiving ART with syphilis (CHI+RPR+ART+, group 6). Additionally, we enrolled 23 age-matched HIV-1-negative individuals from the MSM population with high-risk behaviors as healthy controls (HCs, group 7). The inclusion and exclusion criteria for each group were the same as previously described (15). All groups were matched for age. The characteristics of the subjects are presented in Table 1.

**Table 1:** Characteristics of the participants.

**Characteristics**	**Cases (*n*)**	**Age (years)**	**HIV-RNA (copies/ml)**	**CD4^**+**^ T-cells (cells/μl)**	**RPR titer ≥1:8(*n*)**
HCs	23	34.39 ± 9.40	TND	896.03 ± 282.83	0
AHI	34	33.82 ± 7.43	142297.94 ± 371737.44	471.95 ± 104.50^*^, ^†^	0
CHI+ ART–	25	35.80 ± 11.21	116157.72 ± 237206.35	415.11 ± 211.90^*^, ^†^	0
CHI+ART+	24	32.62 ± 7.62	TND	627.13 ± 310.05^*^	0
RPR+	17	33.82 ± 6.58	TND	851.29 ± 289.78	7
CHI+RPR+ART–	16	31.75 ± 6.51	1307788.06 ± 220541.59	371.45 ± 294.84^*^, ^‡^	11
CHI+RPR+ART+	30	32.53 ± 5.83	TND	606.16 ± 182.23^*^, ^‡^, ^ξ^	18

*HCs, Healthy controls; AHI, Acute HIV-1-infected individuals; CHI+ ART–, chronic HIV-1-infected individuals not receiving ART; CHI+ART+, chronic HIV-1-infected individuals receiving ART; RPR+, RPR and TPPA positive individuals without chronic HIV-1 infection; CHI+RPR+ ART–, RPR and TPPA positive individuals with chronic HIV-1 infection without ART; CHI+RPR+ART+, RPR and TPPA positive individuals with chronic HIV-1 infection receiving ART. Data are presented as the means ± standard deviation. TND, target not detected. RPR, rapid plasma reagin. TPPA, T. pallidum particle agglutination assay*.

* *p < 0.05 vs. CD4^+^ T cell counts of healthy controls*;

† *p < 0.05 vs. CD4^+^ T cell counts of CHI+ART+ individuals*;

‡ *p < 0.05 vs. CD4^+^ T cell counts of RPR^+^ individuals*;

ξ *p < 0.05 vs. CD4^+^ T cell counts of CHI+RPR+ART+ individuals*.

### Cell Surface Staining

**Figure 1:**
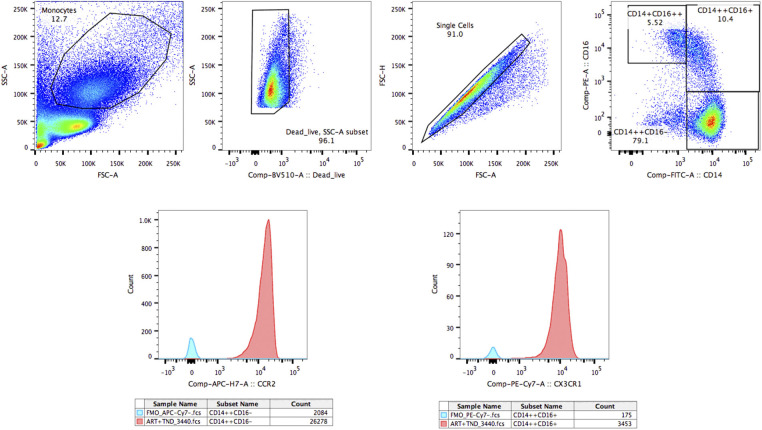
The gating strategy for CCR2 and CX3CR1 on three monocyte subsets. The gating strategy for CCR2 and CX3CR1 on classical monocyte subsets (CD14++CD16–), intermediate monocyte subsets (CD14++CD16+), and non-classical monocyte subsets (CD14+CD16++).

Cryopreserved peripheral blood mononuclear cells (PBMCs) were used, and cell viability was evaluated. Cryopreserved PBMCs were thawed in RPMI 1640 medium (Invitrogen, Carlsbad, CA, USA), washed with PBS containing 1% BSA, and then incubated at room temperature for 20 min with the cell viability fixable viability stain 510 (BD Biosciences, San Jose, CA, USA). The viability of the PBMCs in the study is above 90%, as is shown in Figure 1. Cell surface staining were performed as previously described (15). After stained with anti-CD14-FITC (eBioscience), anti-CD16-PE (eBioscience), anti-CX3CR1-PE-Cy7 (eBioscience Inc., San Diego, CA) and anti-CCR2-APC-Cy7 (Biolegend Inc., San Diego, CA), monocyte phenotypes were detected by flow cytometry using a BD FACSCanto™ II with Diva software (BD Biosciences, San Jose, CA, USA). Then, data were analyzed by using FlowJo 10.0.7 software (Tree Star Inc., Ashland, OR, USA).

### CD4^+^ T-Cell Counts and HIV-1 Viral Load Measurements

CD4^+^ T-cell counts were determined by three-color flow cytometry after stained with anti-CD3-APC, anti-CD4-FITC, and anti-CD8-PE monoclonal antibodies (BD Biosciences). The data were analyzed with a BD FACSCanto™ II Flow Cytometry System (BD Biosciences, San Jose, CA, USA). HIV-1 viral loads were measured with an automated real-time PCR-based m2000 System (Abbott Molecular Inc., Des Plaines, IL, USA).

### Statistical Analysis

Normal distribution and variance homogeneity were tested using one-way analysis of variance (ANOVA). If a significant effect was found, *post hoc* comparisons were conducted to evaluate the differences among groups. All reported *p*-values were two-tailed and were considered significant at *p* < 0.05. If the data were not normally distributed, non-parametric test was used for pairwise comparison, and a much lower pairwise type I error rate was used in the study. The difference was considered significant if the *p*-value was below 0.05/6 = 0.0083.

Statistical analysis was performed with GraphPad Prism 6.0 software (San Diego, CA, USA).

## Results

### Characteristics of Study Participants

There were seven groups included in the study. The characteristics of the participants are listed in Table 1. The ages and sexes of the individuals in the seven groups were matched. The viral loads in HCs, CHI+ART+ individuals, and CHI+RPR+ART+ individuals were target not detected (TND). The mean CD4^+^ T cell counts in HCs were significantly higher than those in other groups except in RPR+ individuals. The mean CD4^+^ T cell counts in CHI+ART+ individuals were higher than those in AHI individuals and in CHI+ ART– individuals, the CD4^+^ T cell counts were higher in RPR+ individuals than those in CHI+RPR+ ART– individuals and CHI+RPR+ART+ individuals. Compared with those in CHI+RPR+ART+ individuals, the mean CD4^+^ T cell counts were lower in CHI+RPR+ ART– individuals.

### Perturbations of CCR2 and CX3CR1-Expressing Monocyte Subsets During HIV-1 and *T. pallidum* Coinfection

The perturbations of three monocyte subsets during HIV and/or *T. pallidum* infection were characterized by our group (14, 15). The perturbations of CCR2 and CX3CR1-expressing monocyte subsets in the study were shown in Table 2, and the results were similar to our previous reports.

**Table 2:** The perturbations of CCR2 and CX3CR1 expressing monocyte subsets.

	**Classical**	**Intermediate**	**Non-classical**
**Groups**	**monocyte subsets**	**monocyte subsets**	**monocyte subsets**
HCs	89.94 ± 3.23	5.59 ± 2.03	5.47 ± 3.45^&^
AHIs	82.21 ± 9.22^#^, ^&^	9.50 ± 4.32^#^, ^&^	8.28 ± 6.87^&^
CHIs+ ART–	85.75 ± 5.12^#^	9.66 ± 4.16^#^, ^&^	4.91 ± 1.89^&^
CHIs+ART+	90.13 ± 5.48^*^, ^†^	7.08 ± 2.47^#^, ^*^, ^†^	4.46 ± 3.33
RPRs+	93.31 ± 2.43^#^	4.28 ± 1.53^#^	3.59 ± 1.70
CHI+RPRs+ ART–	86.35 ± 4.92^#^, ^‡^	10.11 ± 4.21^#^, ^‡^	3.54 ± 3.17
CHI+RPRs+ART+	91.64 ± 2.81^‡^, ^ξ^	5.01 ± 1.94^ξ^, ^&^	3.35 ± 1.55^#^

*HCs, Healthy controls; AHI, Acute HIV-1-infected individuals; CHI+ ART–, chronic HIV-1-infected individuals not receiving ART; CHI+ART+, chronic HIV-1-infected individuals receiving ART; RPR+, RPR and TPPA-positive individuals without chronic HIV-1 infection; CHI+RPR+ ART–, RPR and TPPA-positive individuals with chronic HIV-1 infection without ART; CHI+RPR+ART+, RPR and TPPA-positive individuals with chronic HIV-1 infection receiving ART. Data are presented as the means ± standard deviation. RPR, rapid plasma reagin. TPPA, T. pallidum particle agglutination assay*.

# *p < 0.05 vs. the frequency of monocyte subsets in HCs; p < 0.05 vs. the frequency of monocyte subsets in AHI individuals*;

† *p < 0.05 vs. the frequency of monocyte subsets in CHI+ ART– individuals*;

& *p < 0.05 vs. the frequency of classical monocytes of CHI+ART+ individuals*;

‡ *p < 0.05 vs. the frequency of monocyte subsets in RPR+ individuals*;

ξ *p < 0.05 vs. the frequency of monocyte subsets in CHI+RPR+ ART– individuals*.

The frequency of the intermediate monocyte subsets in AHI individuals was significantly higher than that in healthy controls. The frequency of the classical monocyte subsets was higher in RPR+ individuals than in HCs and CHI+RPR+ patients. In addition, the frequency of the classical monocyte subsets was higher in RPR+ individuals and was lower in CHI+RPR+ ART– individuals than in CHI+RPR+ART+ individuals. The frequency of the intermediate monocyte subsets was higher in CHI+ART+ and CHI+RPR+ ART– individuals than in CHI+RPR+ART+ individuals. The frequency of the non-classical monocyte subsets in CHI+RPR+ART+ individuals was lower than that in HCs.

### CCR2 Density on Monocyte Subsets Decreases During HIV-1 and *T. pallidum* Coinfection Except in AHI Individuals

The gating strategy for CCR2 on monocyte subsets is shown in Figure 1.

**Figure 2:**
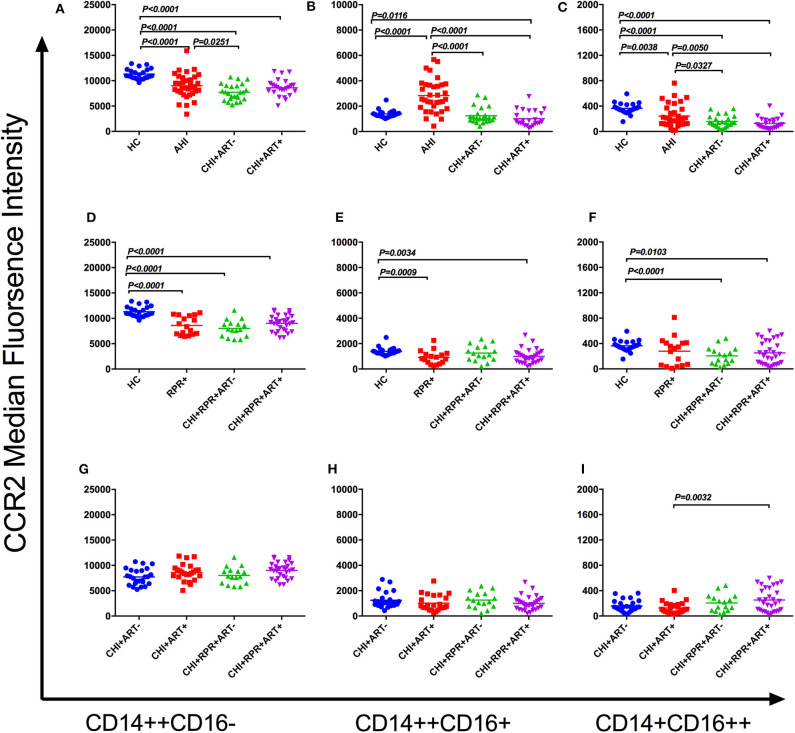
CCR2 perturbations on three monocyte subsets during HIV-1/*Treponema pallidum* coinfection. The median fluorescence intensity (MFI, density) of surface CCR2 on the classical monocyte subsets (CD14++CD16–) **(A)**, intermediate monocyte subsets (CD14++CD16+) **(B)**, and non-classical monocyte subsets (CD14+CD16++) **(C)** in HCs, AHI, CHI+ART–, and CHI+ART+ individuals. The MFI of surface CCR2 on the classical monocyte subsets **(D)**, intermediate monocyte subsets **(E)**, and non-classical monocyte subsets **(F)** in HCs, RPs+, CHI+RPR+ART–, and CHI+RPR+ART+ individuals. The MFI of surface CCR2 on the classical monocyte subsets **(G)**, intermediate monocyte subsets **(H)**, and non-classical monocyte subsets **(I)** in CHI+ ART–, CHI+ART+, CHI+RPR+ART–, CHI+RPR+ART+ individuals. The differences among groups were analyzed by one-way analysis of variance (data with normal distribution and variance homogeneity) or non-parametric test (data without normal distribution), and the differences were considered significant if *p* < 0.05 or *p* < 0.05/6 = 0.0083 (non-parametric test). The solid line indicates the mean or median value.

The median fluorescence intensity (MFI) of CCR2 on the classical monocyte subsets (CD14^++^CD16^−^) in HCs was significantly higher than that in AHI, CHI+ ART– and CHI+ART+ individuals. In addition, the MFI of CCR2 decreased in the CHI+ ART– group compared to that in the AHI group (Figure 2A). The MFI of CCR2 density in HCs was higher than that in RPR+, CHI+RPRs+ ART– and CHIs+RPR+ART+ individuals (Figure 2D).

For CCR2 density on the intermediate monocyte subsets (CD14++CD16+), the MFI of CCR2 in AHI individuals was significantly higher than that in HC, CHI+ ART– and CHI+ART+ individuals, and the density of CCR2 in CHI+ART+ individuals was lower than that in HCs (Figure 2B). The MFI of CCR2 in RPR+ and CHI+RPR+ART+ individuals was lower than that in HCs (Figure 2E).

The MFI of CCR2 on the non-classical monocyte subsets (CD14^+^CD16^++^) in HCs was significantly higher than that in AHI, CHI+ ART–, CHI+ART+. In addition, the CCR2 density in AHI individuals was higher than that in CHI+ ART– and CHI+ART+ individuals (Figure 2C). The CCR2 density was higher in HCs than in CHI+RPR+ ART– individuals and CHI+RPR+ART+ individuals (Figure 2F). The CCR2 density in CHI+ART+ individuals was lower than that in CHI+RPR+ART+ individuals (Figure 2I).

The association among CCR2 expression on the three monocyte subsets and viral loads and CD4^+^ T cell counts was evaluated by Spearman's correlation test. CCR2 density on the intermediate monocyte subsets was inversely correlated with CD4^+^ T cell counts in the CHI+ ART– group (*r* = −0.4237, *p* = 0.0348).

### CX3CR1 Density on Monocyte Subsets Increases During HIV-1 and *T. pallidum* Coinfection

The gating strategy for CX3CR1 on monocyte subsets is shown in Figure 1.

**Figure 3:**
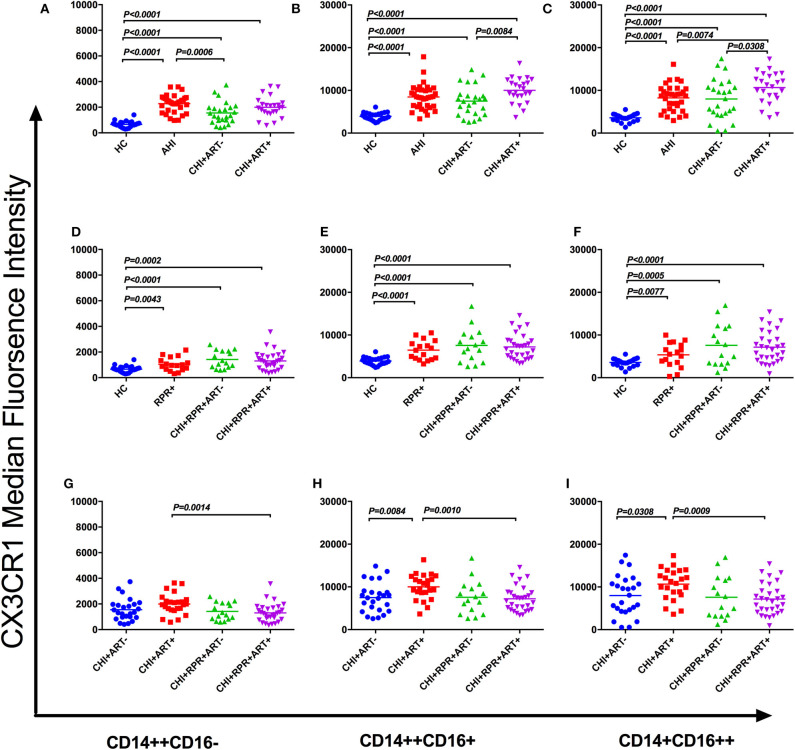
CX3CR1 perturbations on three monocyte subsets during HIV-1/*Treponema pallidum* coinfection. The median fluorescence intensity (MFI, density) of surface CX3CR1 on classical monocyte subsets (CD14++CD16–) **(A)**, intermediate monocyte subsets (CD14++CD16+) **(B)**, and nonclassical monocyte subsets (CD14+CD16++) **(C)** in HCs, AHIs CHI+ART–, and CHI+ART+ individuals. The MFI of surface CX3CR1 on classical monocyte subsets **(D)**, intermediate monocyte subsets **(E)**, and non-classical monocyte subsets **(F)** in HCs, RPR+, CHI+RPR+ART–, and CHI+RPR+ART+ individuals. The MFI of surface CX3CR1 on classical monocyte subsets **(G)**, intermediate monocyte subsets **(H)**, and non-classical monocyte subsets **(I)** in CHI+ ART–, CHI+ART+, CHI+RPR+ART–, CHI+RPR+ART+ individuals. The differences among groups were analyzed by one-way analysis of variance (data with normal distribution and variance homogeneity) or non-parametric test (data without normal distribution), and the differences were considered significant if *p* < 0.05 or *p* < 0.05/6 = 0.0083 (non-parametric test). The solid line indicates the mean or median value.

On the classical monocyte subsets, the density of CX3CR1 was significantly higher in AHI, CHI+ ART– and CHI+ART+ individuals than in HCs, and the density of CX3CR1 in AHI individuals was higher than that in CHI+ ART– individuals (Figure 3A). The density of CX3CR1 on the intermediate and non-classical monocyte subsets was significantly higher in AHI, CHI+ ART– and CHI+ART+ individuals than in HCs, and CX3CR1 density on these two subsets was higher in CHI+ART+ individuals than in CHI+ ART– individuals (Figures 3B,C). The CX3CR1 density on the non-classical monocyte subsets was higher in CHI+ART+ individuals than in AHI individuals (Figure 3C).

As shown in Figures 3D–F, the CX3CR1 density on the three monocyte subsets was significantly lower in HCs than in RPR+, CHI+RPR+ ART–, and CHI+RPR+ART+ individuals. According to Figures 3G–I, the density of CX3CR1 on three monocyte subsets was significantly lower in CHI+RPR+ART+ individuals than in CHI+ART+ individuals, and the density of CX3CR1 on the intermediate monocyte subsets and the non-classical monocyte subsets in CHI+ ART– individuals was lower than that in CHI+ART+ individuals (Figures 3H,I).

## Discussion

In this study, we evaluated the changes of CCR2 and CX3CR1 expression on three monocyte subsets at the different stages of HIV-1/*T. pallidum* coinfection. We found perturbations of CCR2 and CX3CR1 expression on monocyte subsets at the different stages of HIV-1/*T. pallidum* coinfection. We found that CCR2 was downregulated on the classical monocyte subsets but upregulated on the intermediate monocyte subsets during acute HIV-1 infection. The density of CCR2 in RPR+ individuals decreased on the classical and intermediate monocyte subsets, and the density of CCR2 in CHI+ART+, CHI+RPR+ART+ individuals on three monocyte subsets decreased. We also found that CX3CR1 was upregulated on the three monocyte subsets in all groups compared to that on the monocyte subsets in HCs. Compared to that in CHI+ ART– individuals, the density of CX3CR1 in CHI+ART+ individuals was higher on the intermediate and non-classical monocyte subsets, and the density of CX3CR1 in CHI+ART+ individuals was higher than the density of CX3CR1 in CHI+RPR+ART+ individuals on the three monocyte subsets.

The decreased densities of CCR2 expression on the classical monocyte subsets during HIV-1/*T. pallidum* coinfection, which reflects the impaired phagocytosis and chemotaxis of monocytes, may impact the death of infected progenitor cells in the bone marrow. The increased expression of CX3CR1 on monocytes implies systemic inflammation during HIV-1/*T. pallidum* coinfection.

In response to CCL2, CCR2-mediated monocyte recruitment is essential for defense against microbial pathogens (22). CX3CR1 on monocytes results in differentiation into resident tissue cells and is involved in tissue homeostasis (23).

Classical monocyte subsets are involved mostly in preventing pathogen invasion, the intermediate monocyte subsets are involved principally in antigen presentation and inflammatory responses, and the non-classical monocyte subsets are involved mostly in immune surveillance (24). In response to microbial stimuli, inflammatory monocytes secrete CCL2 and traffic to sites of microbial infection via CCR2-mediated emigration to defend against bacteria (25). It was reported that monocytes recruited via CCL2/CCR2, were related to immune activation and inflammation to propagate inflammation and tissue damage in osteoarthritis (OA) (26). Gama et al. demonstrated that CCR2 was downregulated on the classical monocyte subsets during acute HIV-1 infection (27), which was consistent with our findings. In our study, decreased CCR2 expression on the classical monocyte subsets was found in AHI, CHI+ ART– and CHI+ART+ individuals, which indicates impaired chemotaxis. Decreased CCR2 expression may be responsible for the death of infected progenitor cells in the bone marrow which results in a reduction in classical monocyte subsets migrating to the peripheral blood (4, 27) Compared with AHI individuals, there was more CCR2 deficiency in the CHI individuals, which may be responsible for impaired monocyte migration in CHI individuals (28). In this study, we found that CCR2 expression on the intermediate monocyte subsets was higher in AHI individuals than that in HCs, CHI+ ART– and CHI+ART+ individuals. Compared with that in HCs, lower CCR2 expression on monocytes has been found in elite controllers and individuals with suppressed viremia after ART (29).

Our group recently found that the frequency of the classical monocyte subsets increased during syphilis infection (15). However, in this study, the expression of CCR2 on the classical monocyte subsets decreased in RPR+ individuals. Lower proportions of CCR2-expressing cells may reflect increased systemic exposure to their ligand CCL2, causing impaired monocyte migration (28). The levels of CX3CR1 are higher on the non-classical monocyte subsets compared to intermediate monocyte subsets (30). CX3CR1 is required for cellular transendothelial migration and entry into atherogenic plaques, which is associated with cardiovascular disease (CVD) (31). In this study, we found increased expression of CX3CR1 on the three monocyte subsets in HIV-1-infected patients. The increased expression of CX3CR1 on monocytes has been associated with systemic inflammation during HIV-1 infection (4). Although combination antiretroviral therapy (ART) is effective at suppressing HIV viremia to undetectable levels in peripheral blood, HIV-associated inflammation and innate immune activation persists in HIV-1-infected patients with virologic suppression (32). Elite controllers (individuals with suppressed viremia without ART) express higher CX3CR1 levels on monocytes than HIV-negative controls (29). Compared with treated HIV-1 patients, HIV-1 patients with ART initiation have higher CX3CR1 expression on CD16^+^ monocytes (28). Circulating memory CD8^+^ T cells that express CX3CR1 are enriched in HIV-infected recipients after ART, CX3CR1^+^ CD8^+^ T cells could interact with coagulation elements, CX3CR1^+^ CD8^+^ T cells may be associated with CVD risk in HIV-infected ART recipients (33). High levels of CX3CR1 on the intermediate monocyte subsets and non-classical monocyte subsets were found in the study, which may be a risk factor for the development of CVD, perhaps because of the persistent innate immune activation regardless of ART.

In our study, we found increased expression of CX3CR1 on the three monocyte subsets in RPR+, CHI+RPR+ ART– and CHI+RPR+ART+ individuals. It is of interest that the expression of CX3CR1 on the three monocyte subsets was lower in CHI+RPR+ART+ individuals than that in CHI+ART+ individuals. The limited surface antigenicity of *T. pallidum* is poorly detected by the innate immune system, and may promote the evasion of adaptive immune responses (34).

In summary, we evaluated the alterations of CCR2 and CX3CR1 expression on three monocyte subsets during different stages of HIV-1/*T. pallidum* coinfection. Our findings provide new insight into the roles of CCR2 and CX3CR1 on three monocyte subsets in HIV-1 and *T. pallidum* pathogenesis.

## Data Availability Statement

The datasets generated for this study are available on request to the corresponding author.

## Ethics Statement

The studies involving human participants were reviewed and approved by Beijing Youan Hospital Research Ethics Committee. The patients/participants provided their written informed consent to participate in this study.

## Author Contributions

LL, BS, and TZ conceived the study, designed the experiments, and analyzed the data. NG, YC, XY, QZ, and TS performed the experiments. HW, CL, and TZ contributed to reagents and materials. NG, YC, BS, and LL wrote the article. All authors read and approved the final manuscript.

## Conflict of Interest

The authors declare that the research was conducted in the absence of any commercial or financial relationships that could be construed as a potential conflict of interest.
